# Cthrc1 lowers pulmonary collagen associated with bleomycin‐induced fibrosis and protects lung function

**DOI:** 10.14814/phy2.13115

**Published:** 2017-03-14

**Authors:** Andrew P. Binks, Megyn Beyer, Ryan Miller, Renee J. LeClair

**Affiliations:** ^1^ Department of Biomedical SciencesSchool of Medicine, GreenvilleUniversity of South CarolinaGreenvilleSouth Carolina; ^2^ Department of Biomedical SciencesCollege of Osteopathic MedicineUniversity of New EnglandBiddefordMaine

**Keywords:** Collagen, Cthrc1, interstitial lung disease, lung fibrosis

## Abstract

Idiopathic pulmonary fibrosis (IPF) involves collagen deposition that results in a progressive decline in lung function. This process involves activation of Smad2/3 by transforming growth factor (TGF)‐*β* and Wnt signaling pathways. Collagen Triple Helix Repeat‐Containing‐1 (Cthrc1) protein inhibits Smad2/3 activation. To test the hypothesis that Cthrc1 limits collagen deposition and the decline of lung function, Cthrc1 knockout (Cthrc1^−/−^) and wild‐type mice (WT) received intratracheal injections of 2.5 U/kg bleomycin or saline. Lungs were harvested after 14 days and Bronchoalveolar lavage (BAL) TGF‐*β*, IL1‐*β*, hydroxyproline and lung compliance were assessed. TGF‐*β* was significantly higher in Cthrc1^−/−^ compared to WT (53.45 ± 6.15 ng/mL vs. 34.48 ± 11.05) after saline injection. Bleomycin injection increased TGF‐*β* in both Cthrc1^−/−^ (66.37 ± 8.54 ng/mL) and WT (63.64 ± 8.09 ng/mL). Hydroxyproline was significantly higher in Cthrc1^−/−^ compared to WT after bleomycin‐injection (2.676 ± 0.527 *μ*g/mg vs. 1.889 ± 0.520, *P* = 0.028). Immunohistochemistry of Cthrc1^‐/‐^ lung sections showed intracellular localization and activation of *β*‐catenin Y654 in areas of tissue remodeling that was not evident in WT. Lung compliance was significantly reduced by bleomycin in Cthrc1^−/−^ but there was no effect in WT animals. These data suggest Cthrc1 reduces fibrotic tissue formation in bleomycin‐induced lung fibrosis and the effect is potent enough to limit the decline in lung function. We conclude that Cthrc1 plays a protective role, limiting collagen deposition and could form the basis of a novel therapy for pulmonary fibrosis.

## Introduction

Idiopathic pulmonary fibrosis (IPF) results in disruption of the pulmonary parenchymal matrix and its permanent replacement with fibrotic tissue. This irreversible transition leads to declining lung function and eventually death through respiratory failure (median survival, 2–3 years after diagnosis) (Ley et al. 2011). Despite improved accuracy of diagnosis with high‐resolution computerized tomography, patients usually present with established disease and thus limited therapeutic options and even novel therapies are only marginally successful (Rafii et al. 2013; Karimi‐Shah and Chowdhury 2015). Previous treatment with corticosteroids does not improve survival rate (Nagai et al. 1999) and combined anti‐inflammatory and immunomodulatory agents fail to produce significant benefit and may actually be deleterious (Raghu et al. 1991, 2012; Collard et al. 2004). Current antifibrotic agents (pirfenidone and nintedanib) slow disease progression, but their mechanism of action is unclear (Kreuter et al. 2015). IPF's poor prognosis through late detection and lack of effective treatment demonstrates the need for improved diagnostics and specifically targeted therapies.

Recent research suggests that the abnormal extracellular matrix of IPF may be due to aberrant activation and differentiation of fibroblasts, rather than exclusively due to proinflammatory mechanisms (King et al. 2011). This is consistent with the limited success of purely anti‐inflammatory therapies and illustrates the need to also focus research on the fibro‐proliferative response. Without a clear understanding of the molecular signature of the disease and a way of determining disease stage, effective therapeutic efforts will be minimal.

Heightened concentrations of transforming growth factor (TGF‐*β*) and members of the canonical Wnt signaling pathway (*β*‐catenin) are correlated with fibrotic process in IPF (Liu et al. 2009; Guo et al. 2012), and conversely, blockade of either attenuates the fibrotic response in animal models (Kang et al. 2008; Zhou et al. 2012). Both TGF‐*β* and *β*‐catenin‐mediated Wnt signaling promote fibroblast proliferation and epithelial‐mesenchymal transition of alveolar epithelium that result in fibrosis (Scotton and Chambers 2007). These two pathways share a common mediator, Smad 2/3, which in turn is regulated by collagen triple helix repeat‐containing‐1 (Cthrc1) protein. Cthrc1 may serve a protective role by inhibiting Smad 2/3 phosphorylation and dramatically reducing TGF‐*β* and possibly *β*‐catenin Wnt‐induced collagen production (LeClair et al. 2007).

We postulate that Cthrc1's upstream regulation of fibroblast activity gives it the therapeutic potential to stop the progression of IPF. Precedent for this has recently been found in other tissues. In has previously been shown that Cthrc1 inhibits collagen formation in response to vascular damage (Pyagay et al. 2005; Durmus et al. 2006; LeClair and Lindner 2007) and skin repair (Durmus et al. 2006; Li et al. 2011), and that it represents part of a unique molecular signature in human IPF patients (Bauer et al. 2015).

This study used Cthrc1 knockout mice to investigate the role of Cthrc1 in tissue remodeling and test the hypothesis that lack of Cthrc1 would result in greater collagen deposition in response to bleomycin‐induced lung injury. Determining Cthrc1's role may allow its use as a biomarker of IPF to improve early detection of the disease and potentially form a therapeutic target.

## Methods

### Animals and protocol

Cohorts were comprised of 12‐week‐old male Cthrc1^−/−^ animals (Stohn et al. 2012) and 129S6/SvEv controls. Cthrc1^−/−^ animals were backcrossed to 129 S6/SvEv as previously described (Stohn et al. 2012). All experiments were carried out following federal animal research guidelines and were approved by the University of New England IACUC. On day 1, mice underwent intratracheal injection of 2.5 U/kg of bleomycin (to induce pulmonary fibrosis as in previous studies (Clark et al. 1980; Lazenby et al. 1990; Savani et al. 2000)), intratracheal injection of sterile saline or were left untreated. Measurements taken and their timeline are described below. Not all measurements could be collected in the same animal as some techniques excluded the use of others. Animal weights and mortality were recorded.

### Hydroxyproline quantification

Lung tissue was harvested from WT and Cthrc1^−/−^ mice after 14 days. Following excision, the tissue was immediately flash frozen in liquid nitrogen. Using a cold mortar and pestle, the tissue was ground into a powder and placed into preweighed tubes and the tubes were reweighed to ensure an accurate measure of tissue weight. Subsequent acid hydrolysis, desiccation, and hydroxyproline quantification were carried out according to methods used by Edwards and O'Brien (1980).

### Lung compliance

The trachea was cannulated with the lungs in situ and the chest wall completely opened. The cannula was attached to a 10 mL syringe and pressure transducer (Validyne, MC1‐3) via a three‐way tap. An initial preinflation (to 14 cmH_2_O) was performed to open closed airways and stretch lung and airway tissue. After being allowed to deflate, lungs were inflated by 1 mL increments from the syringe while simultaneously measuring airway pressure (to 14 cmH_2_O). Changes in pressure and volume were used to generate the inspiratory pressure–volume relationship (Huang) and the compliance slope between atmospheric pressure (0 cmH_2_O) and 14 cmH_2_O was determined by linear regression. The compliance slope was used in subsequent analysis to compare bleomycin and saline‐injected animals.

### Breathing pattern

Breathing parameters were measured in unrestrained mice, using whole body plethysmography (Buxco, St. Paul, MN) 14 days after intratracheal injection. Mice were placed in the plethysmograph chamber for 30 min and data collected in the last 5 min (when exploratory behavior had diminished) was used in analysis. Respiratory parameters (tidal volume and rate) were then calculated (Onodera et al. 1997). Breathing parameters of bleomycin and saline‐injected animals were compared.

### Cytokine assay

Bronchoalveolar lavage (BAL) samples were obtained from WT and Cthrc1^−/−^ mice 14 days posttreatment. BAL samples were assayed for total protein concentration and assayed for Total TGF‐*β* (abcam ab119557) and IL1‐*β* according to manufacture protocols (R&D systems Quantikine ELISA kit MLB00C). Cytokine quantification was determined, using spectrophotometry. Values for bleomycin and saline‐injected animals were compared.

### Histology

Lung tissue was harvested from saline and bleomycin‐treated animals 14 days after injection. Briefly, animals were perfused‐fixed with 4% paraformaldehyde and embedded frozen. Tissue was sectioned at 5 μm for Sirus red staining. Tissue was also used for immunofluorescence, using anti‐ *β*‐catenin phospho‐Y654 antibody (Abcam [1B11]; Alexa 647 labeled secondary) and phospho‐Smad 2 (GeneTex primary antibody GTX54987; secondary FITC Rockland), using previously published methods. Phospho‐Smad immunofluorescence was quantified, using methods previously published (LeClair et al. 2007). Briefly, Image J software (NIH) was used to quantify the number of nuclei and the number of positive nuclei per field of view (40X) and expressed as percent positive cells (*n* = 4; 1000 nuclei/animal).

### Cell culture and Gene expression assay

Mouse lung fibroblasts (MLFs) were harvested from 12‐week‐old male WT animals (129S6/SvEv; *n* = 3) and cells were isolated, using methods similar to those previously described (Seluanov et al. 2010).

Briefly, lungs were flushed with saline prior to excision and transferred to a 10 cm culture dish. The tissue was cut into small pieces prior to incubation with dispase solution at 37°C for 60 min. Cells were pipetted to break clumps free and FBS was added to stop digestion. Cells were spun and washed three times with DMEM/F12 media supplemented with 15% FBS. Cells used for time course assay were between passages 4 and 6.

For bleomycin assay, MLFs were plated at a density of 5 × 10^5^ cells/plate and allowed to adhere overnight before starting the time course. Cells were exposed to 25 mU/mL bleomycin and cultured for a time course of 14 days. RNA was isolated from cells washarvested at days 0, 3, 7, 9, and 14 following bleomycin exposure. RNA was isolated, using Trizol^®^ reagent and cDNA was generated, using Promega reverse transcription system according to manufacturer recommendations. Gene expression assays for Cthrc1 and GAPDH (Mm01163611_m1 and Mm03302249_g1) were purchased from Applied Biosystems and set up according to the assay direction for a 40‐cycle run. Assays were done in triplicate and data were presented as delta‐delta Ct expressed as percent change from day 0.

### Analysis

The group means were calculated for all the histochemical and physiological parameters described above and are reported below. As few animals were used in multiple measurements, correlation analysis was not feasible. Comparison between the genotypes were made on an *a priori basis* using Student t‐tests to test the hypotheses that lack of Cthrc1 results in (1) a greater inflammatory response (higher levels of TGF‐*β* and IL1‐*β*), (2) greater collagen deposition, and (3) greater reduction in lung compliance.

## Results

### Animal weight and mortality

**Figure 1:**
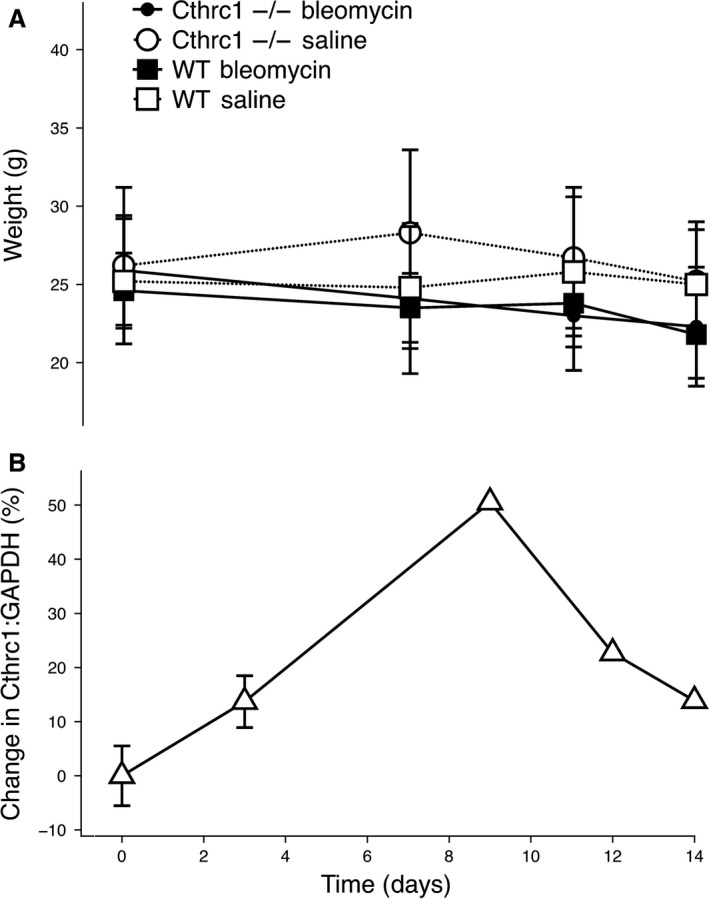
Mean body weight (± standard deviation) of Cthrc1^−/−^ and WT over the 14‐day study period (panel A). Change in expression of Cthrc1in primary mouse fibroblasts following exposure to bleomycin on day 0 (panel B).

Animal weight over the time course of the study is shown in Figure 1, panel A. Eight animals died between days 11 and 14 of the study, and one died on day 7. There was no significant effect of genotype (*P* = 0.719, chi‐square test) or treatment (*P* = 0.408, chi‐square test) on mortality.

### Bleomycin induces Cthrc1 expression in the lung

Cthrc1 gene expression was evaluated in primary mouse lung fibroblasts over a 14‐daytime period following bleomycin exposure. All time points were measured in triplicate and normalized to GAPDH expression and then expressed as change from day 0 (delta‐delta Ct). Cthrc1 expression (Fig. 1, panel B) shows an increase that peaked around day 9 and starting to resolve by day 14.

### Loss of Cthrc1 increases collagen deposition in the lung

**Figure 2:**
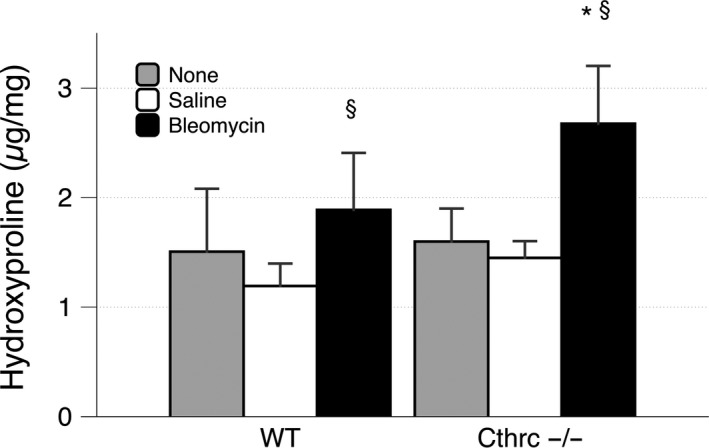
Mean hydroxyproline (± standard deviation) in lung tissue of WT and Cthrc1^−/−^ animals 14 days after no injection (gray) intratracheal injection of saline (white) or bleomycin (black). There was no significant difference between no injection and saline injection in either group. ^§^ indicates a significant difference between saline and bleomycin within a group (WT or Cthrc1^‐/‐^). * indicates a significant difference between groups for a treatment (saline or bleomycin).

Bleomycin caused a significantly greater increase (*P* = 0.028) in hydroxyproline in Cthrc1^−/−^ (2.676 ± 0.527 *μ*g/mg than in WT animals (1.889 ± 0.520 *μ*g/mg, *P* = 0.001), see Figure 2. To validate our saline control, we also determined that there was no significant difference in hydroxyproline in the lungs injected with saline or receiving no injection at all for either the Cthrc1^−/−^ (*P* = 0.293) or WT (*P* = 0.077).

### Fibrotic mediators are elevated in Cthrc1^−/−^ animals

**Figure 3:**
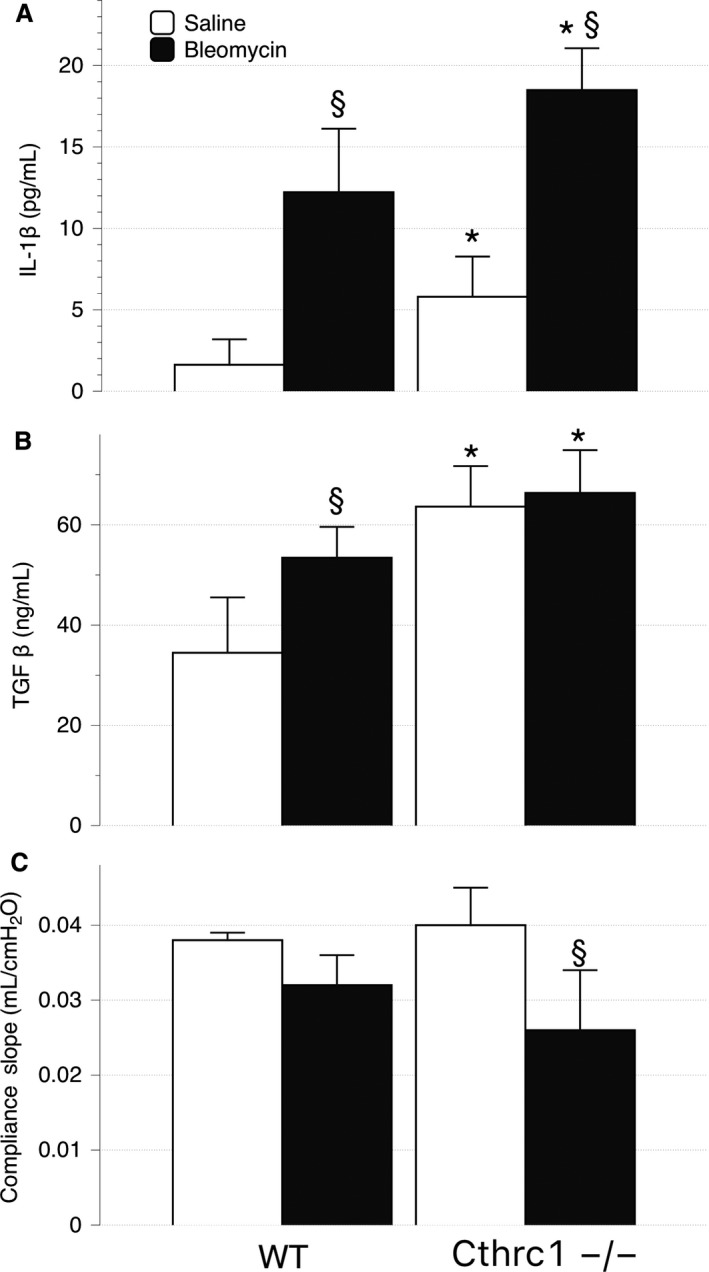
Mean Bronchoalveolar lavage (BAL) fluid levels (± standard deviation) of IL1‐*β* (panel A) and TGF‐*β* (panel B) and in WT and Cthrc1^−/−^ animals 14 days after intratracheal injection of saline (white) or bleomycin (black). The slope of pulmonary compliance curve is shown in panel C. ^§^ indicates a significant difference between treatments (saline and bleomycin) within a group (wild type or Cthrc1^−/−^). * indicates a significant difference between groups for a treatment.

Bleomycin caused significantly higher levels (*P* < 0.001) of IL1‐*β* in Cthrc1^−/−^ (18.49 ± 1.63 pg/mL) than WT (12.22 ± 3.26 pg/mL). Similarly, TGF‐*β* was significantly higher (*P* < 0.001) in Cthrc^−/−^ (66.37 ± 5.16 ng/mL) than WT (53.45 ± 4.4 ng/mL) after bleomycin injection (Fig. 3, panel B). However it should be noted that postsaline levels of both IL1‐*β* and TGF‐*β* (see Fig. 3, panels A and B) were also elevated in Cthrc1^−/−^ mice compared to WT.

### Cthrc1^−/−^ animals have decreased lung function following bleomycin‐induced fibrosis

The rise in hydroxyproline was somewhat reflected in the measures of lung compliance. Bleomycin injection decreased lung compliance in both groups (see Fig. 3, panel C), but there was no statistical difference (*P* = 0.258) between Cthrc1^−/−^ and WT. Changes in compliance were in turn reflected in breathing patterns, with Cthrc1^−/−^ mice adopting the rapid and shallow pattern that is characteristic of advanced fibrotic lung disease (see Table 1).

**Table 1:** Summary of physiological parameters for WT and Cthrc1^−/−^ animals

	Bleomycin		Saline	
	Cthrc 1^−/−^	WT	Cthrc 1^−/−^	WT
Lung Compliance (mL/cmH_2_O)	0.026 ± 0.008 *n* = 6	0.037 ± 0.007 *n* = 7	0.032 ± 0.004 *n* = 4	0.04 ± 0.005 *n* = 2
Tidal Volume (mL)	0.257 ± 0.074 *n* = 9	0.303 ± 0.035 *n* = 5	0.301 ± 0.084 *n* = 11	0.312 ± 0.091 *n* = 10
Respiratory Rate (breaths/min)	282 ± 63 *n* = 9	191 ± 53 *n* = 5	301 ± 115 *n* = 11	274 ± 96 *n* = 10

### Loss of Cthrc1 increases downstream mediators of fibrotic remodeling

**Figure 4:**
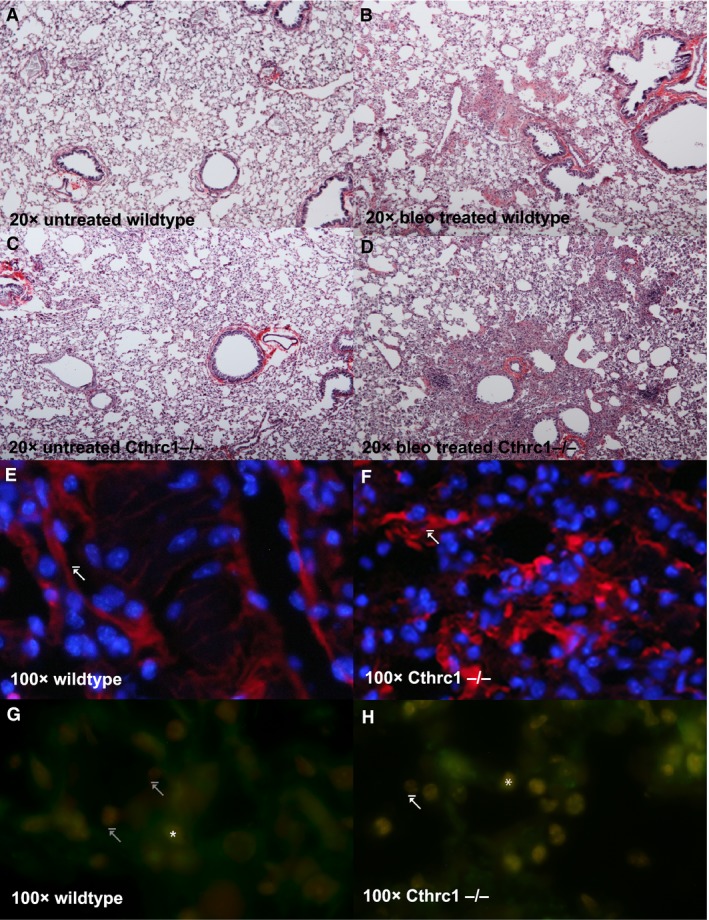
Panels A – D show Sirus red staining of lungs untreated and 14 days post bleomycin treatment. Untreated WT animals (Panel A) and Cthrc1^−/−^ (Panel C) showed similar lung morphology after the 14 day study period. There was a significant increase in collagen deposition in WT (Panel B) and Cthrc1^‐/‐^ (Panel D) 14 days after bleomycin treatment as illustrated by an increase Sirus red staining. Immunofluorescence staining showing localization of *β*‐catenin Y654 in WT (Panel E) and Cthrc1^−/−^ (Panel F) lung tissue harvested 14 days following bleomycin treatment as indicated by arrows. WT animals show a cell membrane restricted localization of the protein consistent in contrast to Cthrc1^−/−^ animals where *β*‐catenin Y654 is no longer restricted to the cell membrane as indicated by the arrows. (Blue: DAPI; Red: anti‐*β*‐catenin phospho‐ Y654 antibody (Abcam [1B11]) visualized with Alexa 647 labeled secondary –TRIC‐labeled secondary). Immunofluroescence staining for pSmad 2 in wild type (Panel G) and Cthrc1^−/−^ (Panel H) lung tissue harvested 14 days following bleomycin treatment. Cthrc^−/−^ showed a significant increase in pSmad2‐positive cells when compared to WT pSmad 2 (Green: GeneTex primary antibody GTX54987 visualized with FITC‐labeled secondary antibody; Red: TOPRO) Representative‐positive cells are indicated with * and negative cells are indicated with an arrow.

Lung collagen accumulation postbleomycin treatment is illustrated by Sirus red staining on lung sections pre‐ and posttreatment. Cthrc1^−/−^ and WT lung sections show similar morphology prior to bleomycin injection (Fig. 4 panels A and C, respectively). Collagen accumulation, shown by an increase in red staining, at day 14 is markedly increased in the Cthrc1^−/−^ when compared to WT (Fig. 4 panels D and B respectively). This is consistent with the increase in collagen as quantified by hydroxyproline analysis.

Immunofluorescence of lung sections at day 14 showed a marked difference in *β*‐catenin phospho‐Y654 localization between the Cthrc1^−/−^ and WT animals (Fig. 4F and E, respectively). In WT animals *β*‐catenin phospho‐ Y654 was localized to vasculature and to a lesser extent in the alveolar epithelium. Specifically, staining was isolated to the cell membrane (indicated by arrow in panel E, Fig. 4). In contrast Cthrc1^−/−^ animals showed significant intracellular levels of *β*‐catenin phospho‐Y654, specifically in areas of advanced remodeling (indicated by arrow panel F, Fig. 4).

At day 14, the percentage of cells that was positive for phospho‐Smad2 expression was significantly greater in Cthrc1^−/−^ than in WT (70.5 ± 15.6% vs. 25.2 ± 10.8%, respectively, *P* < 0.001). Representative immunofluorescence is illustrated in Figure 4G and H; * indicates positive nuclei, arrows indicate negative nuclei.

## Discussion

Our study has demonstrated that Cthrc1 plays a physiologically significant role in lung matrix biology. The absence of Cthrc1 results in an exaggerated response to bleomycin, increased immune response and decreased lung function all characteristics of stereotypical IPF. These mechanical changes manifest in IPF patients as a characteristic rapid, shallow breathing pattern, and severe dyspnea in later stages of the disease. These presenting signs and symptoms are preceded by months of molecular changes (that currently go undetected) as the disease becomes established. The parallel scenario in our model was most likely due to rapid accumulation of collagen. Intratracheal bleomycin injection exacerbated collagen deposition in Cthrc1^−/−^ and produced an IPF phenotype with reduced lung compliance and a consequent shift to a rapid, shallow breathing pattern, consistent with IPF patients. WT animals were affected to a much lesser degree, suggesting Cthrc1 has the ability to mitigate the fibrotic response, and this is most likely mediated through Cthrc1's inhibition of TGF‐*β*/Wnt signaling pathways.

Heightened concentrations of TGF‐*β* and members of the canonical Wnt signaling pathway (*β*‐catenin) have been identified in the lung tissue of pulmonary fibrosis patients (Taylor and du Bois 2001; Konigshoff et al. 2008), suggesting their involvement in the disease pathway. Both TGF‐*β* and *β*‐catenin‐mediated Wnt signaling promote fibroblast proliferation and epithelial to mesenchymal transition (EMT) of alveolar epithelium (Scotton and Chambers 2007) in response to epithelial stress. This response produces a source of myofibroblasts for tissue repair, but unrestrained it may contribute to the deleterious fibrotic process seen in IPF. Systemic suppression of the TGF‐*β*/Wnt pathways as an antifibrotic therapy is unfeasible (knockout of the TGF‐ *β* (Goumans et al. 2009) and Wnt family (Dale 1998) results in lethality), but the TGF‐*β*/Wnt pathways share a common mediator, Smad 2/3. Cthrc1 inhibits Smad 2/3 phosphorylation (activation) and consequently reduces TGF‐*β* (LeClair et al. 2007) and *β*‐catenin Wnt‐induced collagen production. Our data suggests that the moderating action of Cthrc1occurs through, (1) reducing pulmonary fibrosis presumably through its inhibitory effect of both TGF‐*β* (LeClair et al. 2007) and canonical Wnt signaling (Yamamoto et al. 2008), and (2) directly or indirectly mediating inflammation.

### Cthrc1 and TGF‐β initiated fibrosis

Our study shows the absence of Cthrc1 results in an increase in TGF‐*β* in BAL fluids independent of treatment (saline or bleomycin), and this increase is accompanied by an increase in collagen deposition. The substantially elevated postsaline TGF‐*β* likely explains the lack of any further significant increase with bleomycin exposure in the Cthrc1^−/−^ mice, and is consistent with the relatively smaller increase in hydroxyproline after bleomycin exposure compared to WT.

Increasing TGF‐*β* promotes fibrosis via both Smad and non‐Smad pathways. The Smad pathway is initiated via TGF‐*β* binding and autophosphorylation of the Smad 2/3 receptor complex, resulting in its translocation to the nucleus and transcription of target genes. Cthrc1's ability to inhibit Smad 2/3 phosphorylation gives it the potential to reduce gene expression by either of these pathways. Quantification of pSmad 2 in lung sections 14 days posttreatment showed a significant increase in pSmad 2‐positive cells in the Cthrc1^−/−^ animals. This is consistent with both the increase in collagen and decrease in lung function displayed by the knockout animals. More importantly, our finding is demonstrative of Cthrc1's potential as a TGF‐*β* inhibitor and potential as a therapeutic target for pulmonary fibrosis.

### Cthrc1 and canonical Wnt signaling initiated fibrosis

Previous studies suggest Cthrc1 indirectly inhibits the canonical Wnt pathway (Yamamoto et al. 2008). This is supported by our observation that in the absence of Cthrc1 a downstream product of canonical Wnt signaling (*β*‐catenin phospho‐ Y654) was found within cells in areas of advanced remodeling, compared to wild types where it was found membrane bound in nonremodeling tissue. These location differences can potentially be attributed to changes in phosphorylation status of *β*‐catenin Y654 (Winter et al. 2008) that disrupts the interaction with *β*‐catenin and cell surface adhesion molecules. When this interface is compromised, it facilitates the translocation of *β*‐catenin to the nucleus and further enhances Wnt signaling (Lilien and Balsamo 2005; van Veelen et al. 2011). However, caution and further study might be prudent before it is assumed that Cthrc1's influence on *β*‐catenin‐mediated remodeling is independent of the TGF‐*β‐*driven Smad‐dependent and independent pathways described above.

### Increase in proinflammatory cytokines contribute to increased fibrotic response:

The two main contributors to pulmonary fibrosis are excessive fibroblast proliferation and matrix accumulation. The onset of these processes is usually preceded by an acute inflammatory response. This initial response is marked by local elevation of IL1‐*β* (Mia et al. 2014) that is sustained in the chronically inflamed state and fibrogenesis. Although, known to be involved in the acute phase of inflammation, a direct link between chronic disease and IL1‐*β* is unclear. In contrast, TGF‐*β* is a known mediator of fibrotic remodeling and matrix accumulation. Although the cytokine has been implicated as a key player in the development of fibrosis, exposure of myofibroblasts to IL1‐*β* alone has no profibrotic effect on cellular phenotype. Conversely, IL1‐*β* has been shown to attenuate TGF‐*β* mediated collagen deposition, suggesting that it may have a long‐term antifibrotic effect on certain tissues (Siwik et al. 2000). The elevated postsaline IL1‐*β* seen in our Cthrc1^−/−^ animals may be a response to attenuate the TGF‐*β‐*mediated remodeling processes occurring in the absence of Cthrc1 (Siwik et al. 2000).

### Clinical ramifications

Currently, only two drugs have FDA approval for the treatment of IPF, pirfenidone (an anti‐inflammatory, antifibrotic whose mechanism is unclear) and nintedanib (a tyrosine kinase inhibitor). The efficacy of these drugs has recently been questioned because of the absence of a clinically meaningful biomarker (Karimi‐Shah and Chowdhury 2015). However, Bauer et al.'s (2015) recent comparison of bleomycin‐induced transcriptional changes with transcriptional changes in IPF patients identified Cthrc1 as such a molecular signature. Their comparative study also validated the utility of this model with the human disease process, which is critical for translational aspects of this work.

The data presented here clearly demonstrate that the loss of Cthrc1 is detrimental in pulmonary fibrosis. The ability of Cthrc1 to inhibit both canonical Wnt (Yamamoto et al. 2008) and TGF‐*β* (LeClair et al. 2007) signaling make it an attractive molecular target for a specific antifibrotic treatment. A better understanding of its molecular mechanism will facilitate further development of novel therapies for this otherwise progressive and irreversible disease.

### Summary

The results of this study demonstrate that Cthrc1 plays a significant and physiologically important regulatory role in maintaining lung matrix. Absence of Cthrc1 results in excess collagen deposition and an exacerbated response to bleomycin injection. The overall change in matrix related to the systemic loss of Cthrc1 could have implications for other organ systems especially during stress, disease, or aging. To date, there have been no other reports connecting the loss of Cthrc1 systemic matrix changes in other tissues. Our data are consistent with Cthrc1 suppressing of TGF‐*β* and canonical Wnt‐mediated fibroblast proliferation and epithelial–mesenchymal transition (EMT) and TGF‐*β‐*mediated inflammation. The more specific downstream action of Cthrc1 may make it a more viable therapeutic target to reduce the progression of pulmonary fibrotic disease.

## Conflict of Interest

None declared.
